# The dual-path association between upward social comparison and short video addiction: parallel mediating roles of negative emotions and psychological resilience

**DOI:** 10.3389/fpsyg.2026.1797909

**Published:** 2026-05-19

**Authors:** Yuan Tian, Xianli Wang

**Affiliations:** 1 School of Digital Economy and Finance, Guizhou University of Commerce, Guiyang, Guizhou, China; 2 Department of Art and Media Convergence, The Catholic University of Korea, Bucheon, Gyeonggi-do, Republic of Korea; 3 International College, Krirk University, Bangkok, Thailand

**Keywords:** college students, negative emotions, psychological resilience, short video addiction, upward social comparison

## Abstract

**Background:**

Short video addiction among college students has attracted increasing attention. Although upward social comparison has been linked to short video addiction, it remains unclear how negative emotions and psychological resilience jointly explain this association, and whether relevant pathways differ by gender. Drawing on Compensatory Internet Use Theory and the Interaction of Person-Affect-Cognition-Execution model, this study examined a dual-path framework linking upward social comparison to short video addiction through negative emotions and psychological resilience.

**Methods:**

Questionnaire data were collected from 714 college students in China and analyzed using partial least squares structural equation modeling. The model tested the direct association between upward social comparison and short video addiction, the parallel mediating roles of negative emotions and psychological resilience, and gender differences in structural paths.

**Results:**

Upward social comparison was positively associated with short video addiction and negative emotions, and negatively associated with psychological resilience. Both negative emotions and psychological resilience significantly mediated the association between upward social comparison and short video addiction. The indirect association through negative emotions was slightly stronger than that through psychological resilience. Multi group analysis showed that the association between upward social comparison and negative emotions was stronger among female students.

**Conclusion:**

These findings suggest that upward social comparison, negative emotions, psychological resilience, and short video addiction can be understood within a parallel framework of risk related and protective processes. The results highlight the importance of addressing both emotional distress and resilience related resources in college students.

## 1 Introduction

Short video platforms expose users to an almost continuous stream of algorithmically selected and highly idealized content, creating an environment in which upward social comparison may become both frequent and difficult to disengage from. In such environments, users are not only entertained but are also repeatedly confronted with others’ curated appearances, achievements, and lifestyles, which may be associated with greater self-evaluative pressure and a stronger tendency to remain engaged with the platform (Wang et al., 2024). This raises an important psychological question: why might upward social comparison in algorithm-driven short video environments be especially likely to be associated with addictive tendencies? In the present study, short video addiction refers to self-reported problematic short video use characterized by addiction-like features, rather than a clinical diagnosis of behavioral addiction, including difficulty controlling viewing time, reduced impulse control, and disruptions to study and daily life (Zhao and Kou, 2024). Yet, beyond documenting the popularity and social influence of short video platforms, prior discussions have offered limited explanation of the psychological processes through which platform use is associated with addictive tendencies. In particular, the role of upward social comparison in this process remains insufficiently clarified.

Existing research suggests that upward social comparison is not merely a background experience during short video use, but a psychologically salient exposure that may be closely tied to problematic engagement. In algorithm-driven short video environments, users are repeatedly exposed to attractive and socially valued comparison targets during routine browsing, which may intensify self-evaluative pressure and encourage continued attention to platform content (Xiong et al., 2024). This makes upward social comparison theoretically relevant as a focal correlate of short video addiction, not because cross-sectional evidence can establish temporal precedence, but because comparison-related exposure is embedded in the very browsing environment in which problematic use unfolds. At the same time, the psychological processes underlying this association remain insufficiently integrated. One plausible route involves negative emotions (Tian et al., 2023). Compensatory Internet Use Theory (CIU) suggests that when individuals experience distress, they may turn to online activities for short-term relief because online environments offer immediate distraction, low-effort engagement, and rapid affective feedback, all of which may coincide with more frequent and less regulated use (Hu and Huang, 2024). Another plausible route involves protective psychological resources. From the Interaction of Person-Affect-Cognition-Execution (I-PACE) perspective, problematic internet use is associated not only with affective responses but also with person-level resources that shape how individuals cope with comparison-related stress in specific situations (Young and Brand, 2017). Within this framework, psychological resilience represents a key protective resource, and prior work has shown that it is negatively associated with short video addiction (Peng et al., 2025). Thus, the unresolved issue is not whether upward social comparison, negative emotions, and psychological resilience are each relevant in isolation, but whether comparison-related exposure is simultaneously associated with short video addiction through a risk-related emotional route and a protective resource-related route.

Drawing on this gap, the present study integrates CIU and the I-PACE model to examine upward social comparison, negative emotions, psychological resilience, and short video addiction within a single framework. Specifically, this study tests whether negative emotions and psychological resilience are simultaneously associated with the relationship between upward social comparison and short video addiction.

## 2 Theory and hypotheses

### 2.1 Theoretical rationale

This study develops a dual path framework grounded in the I-PACE model and CIU. The I-PACE model conceptualizes problematic internet use as a process linking situational stimuli, affective and cognitive responses, and addiction-related outcomes (Jiang et al., 2025). In the present study, negative emotions are conceptualized as relatively proximal affective responses to upward social comparison, whereas psychological resilience is conceptualized as a relatively stable protective resource within the person domain. Because these two constructs represent different levels of psychological functioning, the current framework does not prespecify a unidirectional temporal sequence between them and instead treats them as conceptually distinct parallel mechanisms. In the context of short video use, upward social comparison may serve as a salient comparison-related stimulus that is associated with stronger negative emotions and lower psychological resilience, which represent distinct affective and personal-resource processes related to a greater tendency toward short video addiction (Zhan and Zhu, 2025). Within this framework, psychological resilience can be understood as a protective psychological resource that is related to more effective coping, emotion regulation, and self-control, whereas lower resilience may be associated with greater difficulty resisting comparison-related stress and uncontrolled use (Mehmood et al., 2025). CIU further complements this perspective by explaining why individuals may turn to short video use for emotional compensation when experiencing stress or negative emotions (Hu and Huang, 2024). Because short video platforms provide immediate feedback and immersive experiences, repeated reliance on them for short-term relief may coincide with higher levels of uncontrolled use and addiction risk (Zhang et al., 2025).

Taken together, the I-PACE model provides the broader process framework, whereas CIU more specifically explains compensatory use driven by negative emotional states. I-PACE can account for how personal characteristics, affective responses, and problematic use are linked, but it does not by itself specify why distress arising from upward social comparison should translate into compensatory short video use. By contrast, CIU is more directly suited to explaining the emotional pathway, but it is less suited to accounting for how protective personal resources operate within the same process. Therefore, the two frameworks are integrated in a complementary rather than redundant way in the present study. Specifically, CIU is primarily used to explain the risk-related emotional pathway, whereas I-PACE provides the broader framework for locating both negative emotions and psychological resilience within the process linking upward social comparison to short video addiction, with psychological resilience positioned within the Person component of I-PACE as a protective psychological resource. In this sense, the present model does not test theoretical competition in a strict sense; rather, it examines the relative salience of a risk-related pathway and a protective pathway within one integrated framework.

### 2.2 Upward social comparison and short video addiction

Upward social comparison refers to the tendency for individuals to evaluate themselves by comparing with others who appear more successful or more idealized (Yuan et al., 2025). Short video platforms primarily feature idealized lifestyles and success-oriented narratives, and algorithmic recommendation systems further reinforce high exposure and repeated viewing contexts. These features provide fertile conditions for the frequent occurrence of upward social comparison (Yu and Zhang, 2025).

Against this background, prior research has increasingly examined the association between upward social comparison and short video addiction. Available evidence suggests that higher levels of upward social comparison are associated with stronger tendencies toward short video addiction (Lewin et al., 2022). This association indicates that upward social comparison is not merely an accompanying experience during platform use. It may also relate to users’ motivations and usage patterns. When users repeatedly encounter representations of others who appear superior, they may shift their browsing behavior toward more frequent, longer lasting, and more engaging use. Such patterns often co-occur with problematic use characteristics, including difficulty controlling use and persistent engagement. Moreover, a systematic review by Xiong et al. (2024) identified upward social comparison as one of the key factors consistently associated with short video addiction across different samples and study designs. When combined, these results imply that an increased risk of short video addiction may be associated with upward social comparison. Such patterns align with the assumptions of CIU (Hu and Huang, 2024). When individuals experience stronger perceptions of self-discrepancy and pressure during social comparison, they may be more inclined to rely on internet use for emotional compensation and temporary relief. Regarding sites for short videos, immediate feedback and immersive information streams facilitate attentional distraction and emotion regulation. Accordingly, individuals reporting higher upward social comparison tend to show more frequent and less regulated usage patterns (Zhang et al., 2025). In light of this theoretical and empirical evidence, the present study advances the following hypothesis: upward social comparison is positively associated with short video addiction.

### 2.3 The mediating role of negative emotions

Negative emotions commonly refer to adverse affective states experienced in daily life, such as depression, anxiety, frustration, and low mood, and they represent typical emotional responses under stressful conditions (Li et al., 2025). Negative emotions are closely associated with academic stress, interpersonal adjustment, and various forms of problematic behavior, and researchers often view them as important risk indicators of mental health. In environments where social media use is highly pervasive, college students may be especially likely to accumulate negative emotions through ongoing processes of social comparison (Lin et al., 2025). Existing evidence can be synthesized into two related claims. First, upward social comparison is consistently associated with stronger negative emotional experiences in digital and social media contexts, including anxiety, rumination, relative deprivation, and related forms of self-focused distress (Jin et al., 2024; Xu and Li, 2024). Second, negative emotions are in turn consistently associated with stronger short video addiction tendencies, and may function as an important linking process between stressful experiences and problematic short video use (Liu et al., 2025; Wang G. Y. et al., 2025). Taken together, these findings support the view that negative emotions are not simply a co-occurring outcome of comparison, but a plausible psychological route through which comparison-related distress may be associated with short video addiction.

According to CIU, when individuals experience higher levels of stress or negative emotional states, they may be more inclined to rely on internet use to obtain emotional compensation and temporary relief (Wang C. G. et al., 2025). On this basis, the present study proposes that upward social comparison is associated with higher levels of negative emotions, and that the accumulation of negative emotions may strengthen the tendency to regulate emotions through compensatory short video use. In this way, negative emotions may represent a potential psychological pathway linking upward social comparison and short video addiction.

Moreover, prior research suggests that women may be more sensitive to external evaluations and appearance-related standards during social comparison, particularly in visually oriented social media environments. For example, a survey study of Chinese college students found that female participants reported greater sensitivity to external appearance standards in social network appearance comparison than male participants (Tong et al., 2024), and related research has shown that appearance-focused social media content may be more strongly associated with negative psychological experiences among women (Pan et al., 2022). In short video contexts characterized by highly curated and appearance-focused content, the positive association between upward social comparison and negative emotions may therefore be stronger among female students than among male students. Accordingly, this study formulates hypotheses regarding the mediating role of negative emotions and gender differences in the emotional pathway.

### 2.4 The mediating role of psychological resilience

Psychological resilience commonly refers to an individual’s capacity to maintain psychological stability and to adapt effectively when facing stress, setbacks, or adversity, and researchers often view it as an important positive psychological resource (Shi et al., 2025). Higher levels of psychological resilience may help individuals respond to environmental challenges more flexibly. Prior work links psychological resilience with better emotion regulation, stronger interpersonal adjustment, and lower levels of problematic behavior (Zhang et al., 2023). In social media contexts, the social comparison environment individuals encounter may closely accompany their psychological resource levels. Prior findings likewise support a resource-based perspective on psychological resilience. In social media contexts, more frequent upward social comparison tends to co-occur with less favorable self-evaluation, weaker coping confidence, and fewer protective psychological resources overall (Johnson, 2021; Li, 2019). At the same time, psychological resilience is consistently negatively associated with short video addiction and related problematic use tendencies, suggesting that individuals with stronger adaptive resources may be less likely to rely on compulsive media use when facing distress (Peng et al., 2025; Wu et al., 2024). This body of evidence makes psychological resilience theoretically relevant not only as a general protective characteristic, but also as a potential pathway linking comparison-related exposure with short video addiction.

From the perspective of the I-PACE model, psychological resources such as psychological resilience represent important individual foundations for problematic internet use. The model emphasizes that differences in personal resources interact with affective and cognitive processing and relate to specific patterns of internet use behavior. On this basis, upward social comparison may be associated with lower psychological resilience, and lower psychological resilience may in turn be associated with higher levels of short video addiction. Within the present framework, psychological resilience is conceptualized as a protective resource-related pathway associated with the link between upward social comparison and short video addiction, rather than as a boundary condition on the emotional pathway. Drawing on the theoretical rationale and prior empirical findings summarized above, we advance the following hypothesis: psychological resilience serves as a mediating pathway linking upward social comparison with short video addiction.

### 2.5 The present study

Although prior research has examined the associations among upward social comparison, negative emotions, psychological resilience, and short video addiction from different perspectives, several limitations remain. Most notably, existing studies tend to explain problematic short video use either from a risk-oriented perspective that emphasizes emotional distress or from a protection-oriented perspective that emphasizes psychological resources. As a result, the literature has not yet clearly resolved whether comparison-related exposure is more meaningfully associated with short video addiction through an emotional risk pathway, a protective resource pathway, or both simultaneously. In addition, many studies have examined upward social comparison, negative emotions, or psychological resilience in isolation, rather than integrating them within a single theory-guided framework. Examining negative emotions and psychological resilience simultaneously therefore contributes not simply by adding another variable, but by clarifying how risk-related and protective processes may coexist, remain distinguishable, and show different levels of salience within the same model.

**Figure 1:**
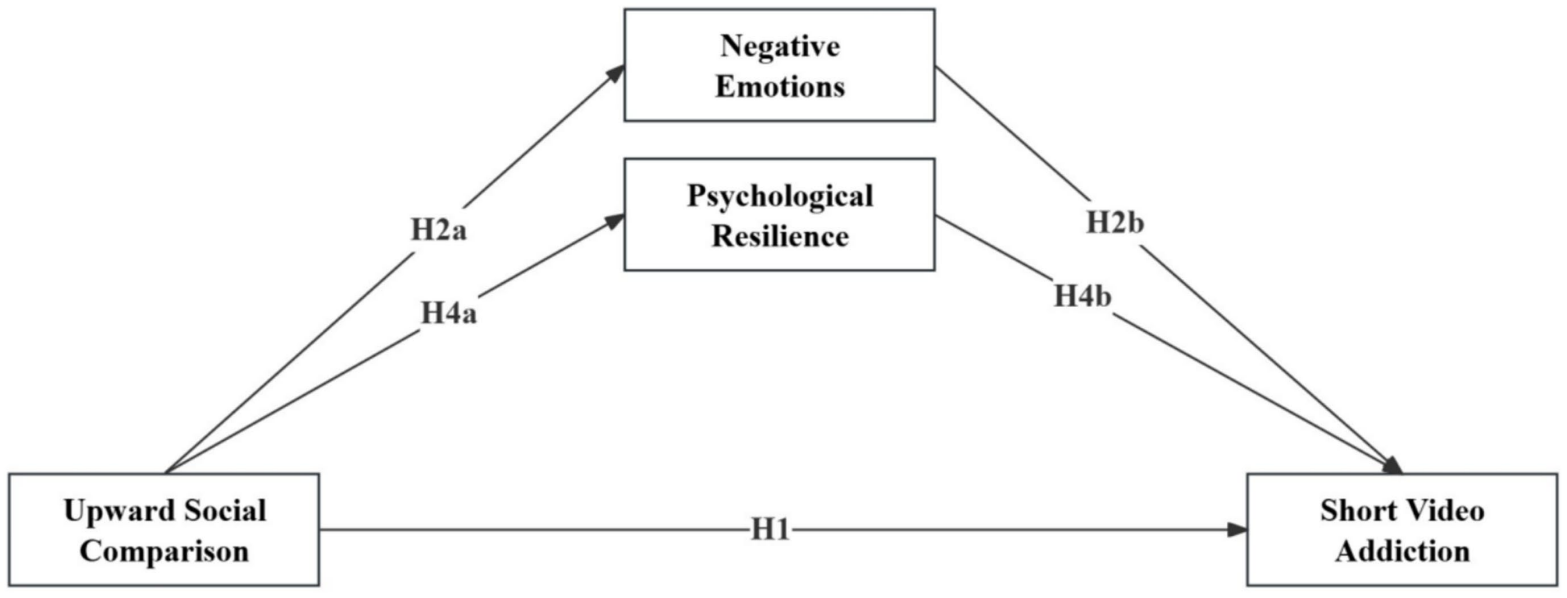
Theoretical hypothesized model.

To address these issues, this study applies the I-PACE model and CIU to specify a parallel mediation framework linking upward social comparison, negative emotions, psychological resilience, and short video addiction. The indirect associations via negative emotions and psychological resilience are examined. The conceptual model is presented in Figure 1, and the hypotheses are outlined accordingly.

*H1*: Upward social comparison is positively associated with short video addiction.

*H2a*: Upward social comparison is positively associated with negative emotions.

*H2b*: Negative emotions are positively associated with short video addiction.

*H3*: Negative emotions mediate the association between upward social comparison and short video addiction.

*H4a*: Upward social comparison is negatively associated with psychological resilience.

*H4b*: Psychological resilience is negatively associated with short video addiction.

*H5*: Psychological resilience mediates the association between upward social comparison and short video addiction.

*H6*: The association between upward social comparison and negative emotions differs by gender, such that this positive association is stronger among female students than among male students.

## 3 Materials and methods

### 3.1 Participants

The study employed a cross-sectional approach and targeted college students enrolled at universities across China. This study was approved by the Ethics Committee of Guizhou University of Commerce. All participants provided informed consent prior to participation in the study. Through university databases and educational collaboration networks, the research team contacted multiple types of undergraduate institutions located in different regions of China. A total of 12 undergraduate universities were purposively selected from six provinces across eastern, central, and western China to ensure geographic diversity. Simple random sampling was then conducted within these institutions, with all students included in the sampling frame, and no institution declined participation. During data collection, designated faculty members at each university informed selected students about the study purpose, confidentiality principles, and voluntary participation requirements. All participants completed the questionnaire independently and anonymously. We conducted the survey between November 2025 and January 2026, and we distributed and collected questionnaires online via the Sojump platform.^1^ Following sample size estimation guidelines, each measurement item required at least ten respondents. The questionnaire included 42 items, and we allowed for an additional attrition rate of approximately 20%. Based on these considerations, the target sample size was set at 504 participants (Kline, 2023). We distributed 755 questionnaires and retrieved all of them. We excluded invalid questionnaires based on the following criteria: (1) more than 20% missing responses, (2) selecting the same extreme option for more than 80% of items, and (3) less than one-third of the median completion time is considered an abnormally low response time (Bowling et al., 2023; Meade and Craig, 2012). 714 valid questionnaires were kept after screening, resulting in an effective response rate of 94.57%. Participation was fully voluntary, non-mandatory, and anonymous, and no incentives were offered. Detailed participant characteristics appear in Table 1.

**Table 1:** Participant demographics (*N* = 714).

Demographic characteristics	Category	Quantity	Percentage (%)
Gender	Female	364	51.0
	Male	350	49.0
Age	18–20	186	26.1
	20–22	167	23.4
	22–24	196	27.5
	> 24	165	23.1
Grade	freshman	199	27.9
	sophomore	159	22.3
	junior	165	23.1
	senior	191	26.8

### 3.2 Measures

#### 3.2.1 Upward social comparison (Iowa Netherlands comparison orientation measure, INCOM)

We measured upward social comparison with the INCOM. Gibbons and Buunk (1999) developed the original version, and Bai et al. (2013) later provided a Chinese revision. The INCOM contains six items, for example, “I often like to compare myself with people who do better than I do.” Participants rated all instruments in this study on a five-point Likert scale. We computed a total score, with higher values reflecting a stronger tendency toward upward social comparison. Prior work has supported the reliability and validity of this measure among Chinese college students (Xu and Li, 2024). Our confirmatory factor analysis suggested an adequate fit (χ^2^/df = 1.586, GFI = 0.996, NFI = 0.997, IFI = 0.999, TLI = 0.997, CFI = 0.999, RMSEA = 0.029).

#### 3.2.2 Short video addiction scale (SVAS)

We assessed short video addiction using the SVAS developed by Qin (2020). The SVAS includes 14 items, such as “I feel restless when I cannot watch short videos.” Participants responded on the same five-point Likert format used throughout the study. Higher total scores indicate more severe self-reported problematic short video use with addiction-like features. Evidence from Chinese college students samples has documented satisfactory reliability and structural validity for this scale (Zhao and Kou, 2024). In this study, confirmatory factor analysis supported a good fit (χ^2^/df = 4.860, GFI = 0.936, NFI = 0.956, IFI = 0.965, TLI = 0.974, CFI = 0.965, RMSEA = 0.074).

#### 3.2.3 Negative emotions

We measured negative emotions with the negative affect subscale of the Positive and Negative Affect Schedule, as revised by Qiu et al. (2008). This subscale consists of nine items, including “ashamed.” Participants rated recent experiences using the five-point Likert response format. Higher scores represent stronger negative emotions. Prior evidence supports the use of this measure among Chinese college students (Liu et al., 2024). Confirmatory factor analysis in our data indicated good model fit (χ^2^/df = 3.284, GFI = 0.990, NFI = 0.996, IFI = 0.997, TLI = 0.989, CFI = 0.997, RMSEA = 0.057).

#### 3.2.4 Psychological resilience (Connor Davidson Resilience Scale 10, CD-RISC-10)

We assessed psychological resilience using the CD-RISC-10 (Campbell-Sills and Stein, 2007). The instrument includes ten items, for example, “I am able to adapt to change.” Participants responded using the same five-point Likert format. Higher total scores reflect higher psychological resilience. In the present study, psychological resilience was conceptualized as a relatively stable, trait-like protective psychological resource rather than a momentary state-like condition. Su and He (2023) reported that this scale meets psychometric standards and is suitable for use in the Chinese context. In the current study, confirmatory factor analysis showed acceptable fit (χ^2^/df = 4.342, GFI = 0.975, NFI = 0.983, IFI = 0.986, TLI = 0.973, CFI = 0.986, RMSEA = 0.068).

## 4 Results

### 4.1 Structural equation modeling analysis

We analyzed the structural model using partial least squares structural equation modeling (PLS-SEM). The measurement model was validated using confirmatory factor analysis in AMOS. This step was necessary to ensure the measurement model fit and justify the methodological choice. The model examined the association between upward social comparison and short video addiction and included four latent constructs measured by 42 items in 714 participants. Considering the model complexity and sample size, PLS-SEM was suitable (Hair et al., 2021).

### 4.2 Measurement model

We evaluated measurement quality following Hair et al. (2021). Outer loadings and composite reliability were used to check reliability. We assessed convergent validity via average variance extracted (Table 2). Discriminant validity was evaluated using the heterotrait monotrait ratio (Table 3) and the Fornell-Larcker criterion (Table 4). Overall, the measurement results met acceptable standards.

**Table 2:** Reliability and validity statistics (*N* = 714).

Constructs	Items	Loadings	Cronbach’α	CR	AVE
USC	USC1	0.792	0.895	0.897	0.657
	USC2	0.815			
	USC3	0.699			
	USC4	0.843			
	USC5	0.855			
	USC6	0.848			
PR	PR1	0.642	0.915	0.918	0.568
	PR2	0.768			
	PR3	0.823			
	PR4	0.671			
	PR5	0.800			
	PR6	0.783			
	PR7	0.810			
	PR8	0.729			
	PR9	0.745			
	PR10	0.742			
NE	NE1	0.823	0.934	0.935	0.657
	NE2	0.885			
	NE3	0.853			
	NE4	0.767			
	NE5	0.699			
	NE6	0.826			
	NE7	0.750			
	NE8	0.796			
	NE9	0.875			
SVA	SVA1	0.727	0.938	0.940	0.555
	SVA2	0.706			
	SVA3	0.713			
	SVA4	0.730			
	SVA5	0.744			
	SVA6	0.737			
	SVA7	0.722			
	SVA8	0.797			
	SVA9	0.753			
	SVA10	0.726			
	SVA11	0.789			
	SVA12	0.751			
	SVA13	0.742			
	SVA14	0.781			

USC, Upward Social Comparison; SVA, Short Video Addiction; PR, Psychological Resilience; NE, Negative Emotions.

**Table 3:** Discriminant validity assessment via HTMT index (*N* = 714).

Constructs	USC	PR	NE	SVA
USC				
PR	0.721			
NE	0.591	0.769		
SVA	0.844	0.819	0.788	

USC, Upward Social Comparison; SVA, Short Video Addiction; PR, Psychological Resilience; NE, Negative Emotions.

**Table 4:** Fornell–Larcker criterion for discriminant validity (*N* = 714).

Constructs	USC	PR	NE	SVA
USC	***0.810***			
PR	−0.667	***0.753***		
NE	0.556	−0.713	***0.810***	
SVA	0.802	−0.763	0.739	***0.745***

USC, Upward Social Comparison; SVA, Short Video Addiction; PR, Psychological Resilience; NE, Negative Emotions; The square root of the AVE value is displayed in bold and italic on the diagonal of the table.

### 4.3 Common method bias and confirmatory factor analysis

We checked potential common method bias using several complementary approaches. The Harman single factor test identified eight factors with eigenvalues above 1, and the first factor explained 36.581% of the total variance, which falls below the 40% criterion (Podsakoff and Organ, 1986). We also added a common latent factor to the measurement model and compared it with the baseline model, and the results showed no significant difference (*p* > 0.05). In addition, confirmatory factor analysis was conducted to evaluate the measurement model, showing acceptable fit: χ^2^/df = 4.537, SRMR = 0.051, GFI = 0.922, NFI = 0.911, IFI = 0.918, TLI = 0.916, CFI = 0.927, and RMSEA = 0.041, consistent with recommended criteria (Hu and Bentler, 1998). Furthermore, we performed a CFA marker test by including a theoretically unrelated marker variable in the measurement model. The model fit indices remained comparable to the baseline model, indicating that the potential impact of common method bias on the results was minimal. Procedural remedies such as anonymous responses and careful item wording were also employed to reduce potential bias.

### 4.4 Structural model

#### 4.4.1 Collinearity assessment

We examined collinearity using variance inflation factors (Hair et al., 2021). All variance inflation factor values were below 3.3 (Table 5), suggesting no notable collinearity issue.

**Table 5:** Collinearity diagnostics for the structural model (*N* = 714).

Constructs	USC	PR	NE	SVA
USC		1.000	1.000	1.845
PR				2.593
NE				2.082
SVA				

USC, Upward Social Comparison; SVA, Short Video Addiction; PR, Psychological Resilience; NE, Negative Emotions.

#### 4.4.2 Significance testing of structural paths

We tested the structural paths in PLS-SEM using bootstrapping with 5,000 resamples. We judged a path as statistically significant when t exceeded 1.96, p was below 0.05, and the 95% confidence interval excluded zero (Hair et al., 2021). All specified paths satisfied these criteria, and the hypotheses were supported (Table 6).

**Table 6:** Structural path estimates and significance tests (*N* = 714).

Relationships	Original sample(O)	2.50%	97.50%	*t*	*p*	Results
USC → PR	−0.667	−0.718	−0.610	24.517	<0.001	Supported
USC → NE	0.556	0.500	0.608	20.173	<0.001	Supported
USC → SVA	0.483	0.424	0.538	16.558	<0.001	Supported
PR → SVA	−0.215	−0.281	−0.157	6.740	<0.001	Supported
NE → SVA	0.317	0.261	0.371	11.264	<0.001	Supported

USC, Upward Social Comparison; SVA, Short Video Addiction; PR, Psychological Resilience; NE, Negative Emotions.

For model performance, short video addiction showed R^2^ = 0.785 and adjusted R^2^ = 0.784, suggesting that the set of predictors had strong explanatory strength for short video addiction. Predictive relevance was also acceptable, with Q^2^ = 0.428 (Table 7).

**Table 7:** Explained variance and predictive relevance (*N* = 714).

Constructs	R^2^	R^2^_Adjusted_	Q^2^
SVA	0.785	0.784	0.428

SVA, Short Video Addiction.

#### 4.4.3 Mediation analysis

**Figure 2:**
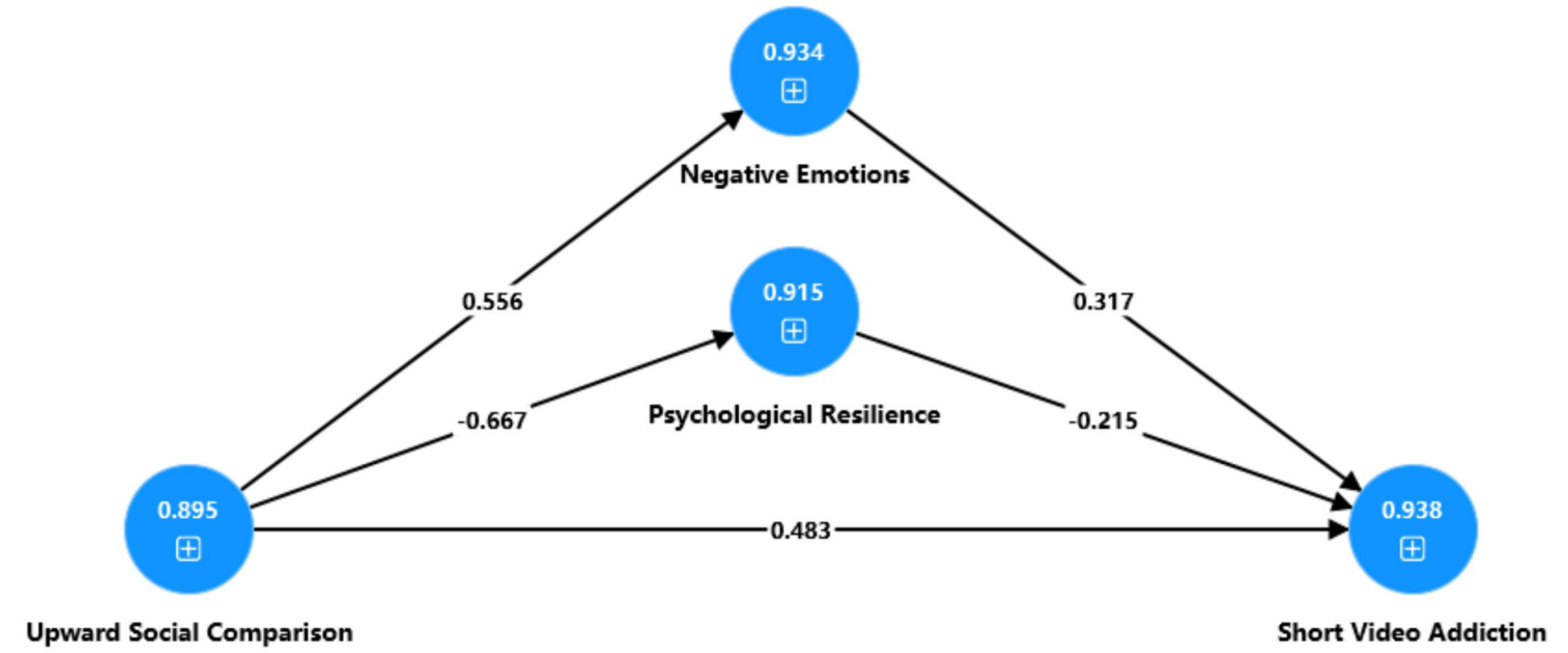
Path diagram of the mediation effect analysis. The numbers in the blue circles denote Cronbach’s alpha coefficients. The numbers on the straight lines indicate path coefficients.

After adjusting for gender, age, and academic grade, the analyses indicated that negative emotions and psychological resilience each carried a significant indirect association between upward social comparison and short video addiction, consistent with partial mediation. The mediation estimates are summarized in Table 8, and Figure 2 presents the corresponding path model.

**Table 8:** Test of mediation effects in the model (*N* = 714).

Path	Indirect Effect	2.50%	97.50%	*t*	*p*	Results
USC → NE → SVA	0.176	0.142	0.212	9.874	<0.001	PM
USC → PR → SVA	0.143	0.101	0.195	6.049	<0.001	

USC, Upward Social Comparison; SVA, Short Video Addiction; PR, Psychological Resilience; NE, Negative Emotions; PM, Partial Mediation.

### 4.5 Multi group analysis

#### 4.5.1 Measurement invariance testing

We examined measurement invariance using the measurement invariance of composite models procedure (Henseler et al., 2016). As shown in Table 9, measurement invariance was established for all constructs except psychological resilience.

**Table 9:** MICOM step 2 results for compositional invariance by gender (*N* = 714).

Constructs	Correlation permutation mean	5% Quantile of empirical distribution of *c_u_*	*p*	Compositional invariance?
USC	1.000	0.998	0.174	Yes
PR	1.000	0.999	0.039	No
NE	1.000	1.000	0.262	Yes
SVA	1.000	0.999	0.116	Yes

USC, Upward Social Comparison; SVA, Short Video Addiction; PR, Psychological Resilience; NE, Negative Emotions.

#### 4.5.2 Gender differences

After establishing measurement invariance, we conducted multi group analysis by comparing structural paths across groups (Zhou et al., 2014). The results indicated significant group differences in the association between upward social comparison and negative emotions, with a stronger association observed among female participants than among male participants. The path coefficient comparisons across gender groups are reported in Table 10.

**Table 10:** Group differences in path coefficients by gender (*N* = 714).

Path	Original (female)	Original (male)	Difference (female–male)	2.50%	97.50%	*p*
USC → NE	0.630	0.330	0.300	0.566	0.680	<0.001

USC, Upward Social Comparison; NE, Negative Emotions.

## 5 Discussion

The results indicated a significant positive link between upward social comparison and short video addiction. This finding supports H1. It suggests that in short video use contexts, individuals who frequently compare themselves with others who appear better off may be more likely to show excessive engagement with and reliance on short video platforms. This pattern aligns with prior research. For instance, prior evidence has linked upward social comparison to multiple manifestations of problematic internet use (Lewin et al., 2022). Similarly, research indicates that when individuals encounter large amounts of idealized self-presentation on social media, they may be more likely to rely on sustained short video use to regulate feelings of imbalance (Xiong et al., 2024). From the perspective of CIU, when individuals perceive unmet needs in real life or experience setbacks in self-evaluation, they may be more inclined to turn to online activities for psychological compensation, which may accompany a higher risk of addictive tendencies (Kardefelt-Winther, 2014).

Our findings indicated that upward social comparison was positively linked to negative emotions, and negative emotions were in turn positively linked to short video addiction. The indirect association through negative emotions was also significant, consistent with a mediating role and supporting H2a, H2b, and H3. Specifically, higher levels of upward social comparison tend to co-occur with more intense negative emotional experiences, and these negative emotions are in turn associated with higher levels of short video addiction. This pattern aligns with evidence reported in earlier research. Earlier studies indicate that, within social media settings, upward social comparison often coincides with reduced self-esteem and elevated depressive symptoms, particularly when individuals are repeatedly exposed to idealized portrayals of others’ lives (Liu et al., 2017). More recent evidence further indicates stable positive associations between upward social comparison and negative emotions such as depression, anxiety, and feelings of frustration (Xu and Li, 2024). Accordingly, under conditions of frequent information exposure on short video platforms, individuals may be more likely to experience self-devaluation, relative deprivation, or a sense of loss when evaluating themselves against more favorable reference standards. These experiences may coincide with elevated levels of negative emotions. At the same time, individuals with higher levels of negative emotions are more likely to report stronger tendencies toward short video addiction (Wang G. Y. et al., 2025). From the perspective of CIU, when individuals are in negative emotional states, online entertainment such as short video use may be more likely to function as a means of emotion regulation and temporary escape. As a result, use intensity and addiction related tendencies often show a closer association (Hu and Huang, 2024). The present findings are more appropriately interpreted as reflecting a general psychological mechanism through which upward social comparison is associated with negative emotions and, in turn, with short video addiction. Within the Chinese cultural context, a strong orientation toward social evaluation and concerns about face may make this mechanism especially salient in everyday short video use by heightening attention to perceived gaps and shortcomings during social comparison (Tong et al., 2024). In this sense, the Chinese cultural context is better understood as a contextual condition that may shape the salience of this pathway in the present sample, rather than as evidence that the underlying mechanism is qualitatively different from that in Western counterparts.

We also identified psychological resilience as a relevant intermediary component in the model. Upward social comparison was negatively linked to psychological resilience, and psychological resilience was negatively linked to short video addiction. The indirect association via psychological resilience was significant as well, supporting H4a, H4b, and H5. These results indicate that in short video use contexts, higher levels of upward social comparison often co-occur with lower psychological resilience, and lower psychological resilience is in turn associated with higher levels of short video addiction. Prior research suggests that frequent upward social comparison is closely linked to weakened positive psychological resources. Repeatedly contrasting the self with others’ idealized images may be associated with lower levels of adaptive and recovery-related psychological resources when facing stress and setbacks (Johnson, 2021). Meanwhile, individuals with higher psychological resilience typically show stronger emotion and behavior self-regulation, and they may be less likely to report short video addiction when encountering negative stimuli (Peng et al., 2025). Within the I-PACE framework, personal traits and psychological resources operate together with situational cues and affective and cognitive responses, and these processes relate to addiction related outcomes. In a short video environment characterized by strong reinforcement and immediate feedback, lower psychological resilience may reflect greater difficulty in maintaining self-control and delay of gratification, which may be associated with more severe short video addiction (Zhang et al., 2024).

Notably, the indirect associations differed across the two parallel pathways. The indirect association through negative emotions (0.176) was slightly larger than that through psychological resilience (0.143). This pattern suggests that in this sample, the association between upward social comparison and short video addiction may more prominently involve compensatory use processes that accompany negative emotional experiences. Specifically, individuals may be more likely to use the immediate entertainment and emotional distraction provided by short videos to regulate the distress triggered by social comparison (Hu and Huang, 2024). At the same time, although psychological resilience also showed a significant pathway, it more closely reflects relatively stable protective resource characteristics. It may therefore capture individual differences in coping and regulatory capacity under stress and setbacks, which may help explain its indirect association with short video addiction.

Consistent with H6, the present study identified a significant gender difference in the association between upward social comparison and negative emotions, with this association being stronger among female participants than among male participants. This finding indicates that the emotional pathway from upward social comparison to negative emotions shows gender-based heterogeneity rather than emerging as a purely exploratory pattern. It suggests that the emotional risk linked to upward social comparison may not operate uniformly across users in short video contexts. Female users may be more likely to experience self-devaluation, anxiety, or frustration when exposed to comparison-related cues. Prior research has shown that women tend to be more sensitive to others’ evaluations and external standards during social comparison processes (Tong et al., 2024). In the present sample, this stronger association may be interpreted as a gendered manifestation of the broader psychological mechanism identified in this study, rather than as a pattern unique to Chinese female students. At the same time, within the Chinese social context, female students may face greater evaluative pressure with respect to appearance, lifestyle, and role performance (Lang and Ye, 2024). When these sociocultural expectations intersect with the frequent presentation of appearance-focused and highly curated lifestyle content on short video platforms, upward social comparison may be more readily linked to negative emotional experiences among female users. From a theoretical perspective, this result further suggests that gender may shape the strength of the emotional risk pathway within the integrated framework, highlighting that the link from upward social comparison to negative emotions is characterized by group heterogeneity rather than a uniform pattern across users (Pan et al., 2022).

## 6 Implications

### 6.1 Theoretical implications

First, building on CIU and the I-PACE model, this study integrates two theoretical dimensions-emotional compensation motives and individual psychological resources-and examines emotional response pathways and psychological resource pathways in parallel within a single model linking upward social comparison and short video addiction. These results advance theory by demonstrating that emotional and resource-based pathways play distinct roles in short video addiction, highlighting the importance of considering both immediate emotional responses and longer-term psychological resources in theoretical models. The findings show that both indirect pathways reach statistical significance, with the indirect association through negative emotions slightly larger than that through psychological resilience. This finding challenges existing models that focus solely on risk or protective factors, showing that both risk-related emotional processes and protective psychological resources must be integrated to fully understand short video addiction.

Second, the multi group analysis revealed a significant gender difference: the link between upward social comparison and negative emotions was stronger for female participants than for male participants. This extends prior work by showing that the emotional association linked to upward social comparison differs across gender groups in short video contexts, thereby providing a more nuanced understanding of individual differences in psychological processes. These results suggest that theoretical models of short video addiction should pay greater attention to group heterogeneity and individual characteristics to more accurately capture the underlying psychological processes.

Third, this study extends prior work by demonstrating how upward social comparison interacts with both emotional and resource-based pathways to influence short video addiction, providing a more nuanced theoretical framework that integrates risk and protective processes. By explicitly modeling parallel mediating pathways, the findings offer clear theoretical contributions to models of problematic short video use and highlight directions for future research on emotional and psychological resource pathways.

Taken together, these findings clarify the study’s contributions: integrating CIU and I-PACE provides a comprehensive framework linking emotional compensation and protective psychological resources; modeling negative emotions and psychological resilience as parallel mediators highlights the coexistence and relative salience of risk and protective pathways; and gender-based differences underscore the importance of considering individual variability in models of problematic short video use.

### 6.2 Practical implications

First, at the student level, the findings suggest the value of guiding college students to increase awareness of upward social comparison and its psychological correlates. Such efforts may help students recognize emotional fluctuations and potential loss of control that accompany comparison during short video use. Mental health courses or group-based programs could focus on fostering more adaptive emotion regulation strategies and strengthening capacities related to psychological resilience, such as coping with stress, self-acceptance, and delay of gratification. These approaches may help reduce reliance on short video use as a primary means of emotional compensation.

Second, at the institutional level, universities may consider integrating short video use guidance with mental health education. Schools could implement structured programs that emphasize emotion management and the development of psychological resilience. For example, orientation programs and psychology courses could incorporate content on social comparison and media literacy to help students understand the selective and idealized nature of platform content. In addition, campus physical activity programs, peer support initiatives, and counseling services may provide diverse channels for emotion regulation and stress relief.

Third, the findings provide practical implications at the platform level. Platforms could refine recommendation mechanisms to limit repeated exposure to highly idealized content and integrate supportive features, such as screen time reminders, emotional state check ins, and healthy use prompts, to facilitate users’ self-monitoring and self-regulation. Increasing the visibility of positive psychological content and representations of real-life diversity may also help attenuate the psychological burden associated with excessive social comparison.

## 7 Limitations and future directions

First, the cross-sectional design restricts conclusions to associations at a single time point. Future research could apply longitudinal or context-based designs to examine whether the observed relationships remain stable across time and usage situations. Second, reliance on self-report measures may not fully reflect actual short video use or momentary emotional experiences. Future studies could integrate questionnaire data with platform-based records, behavioral logs, or experience sampling methods. Third, the sample consisted of Chinese college students, which may limit generalizability. Future research could test the model in other cultural contexts and age groups, especially among older adults. Compared with college students, older adults may differ in emotion regulation patterns, cognitive functioning, and motives for digital media use, which means that the relative salience of the pathways identified in the present study may not be identical across age groups. Fourth, although the present study focused on negative emotions and psychological resilience, other mechanisms may also help explain short video addiction. In particular, self-regulatory resources may represent a more direct and intuitive explanatory pathway. Prior research has suggested that problematic technology use is related to executive control, attention regulation, and other cognitive processes beyond emotional experiences alone (Toh et al., 2023; Wilmer et al., 2017). Future research could therefore further examine self-control, executive function, or delay of gratification as additional explanatory mechanisms.

## 8 Conclusion

Grounded in an integrated CIU and I-PACE framework, this study examined how upward social comparison relates to short video addiction among 714 Chinese college students. The findings indicate that upward social comparison is associated with higher short video addiction, and that this association is jointly carried by elevated negative emotions and reduced psychological resilience, with the negative-emotion pathway slightly stronger. In addition, the link between upward social comparison and negative emotions appears stronger for female students. Together, these results underscore the importance of addressing both emotional distress and resilience-related resources when considering short video addiction in college populations.

## Data Availability

The datasets presented in this study can be found in online repositories. The names of the repository/repositories and accession number(s) can be found in the article/supplementary material.

## Footnotes

1
www.Sojump.com
